# Comparative genomics of cyclin-dependent kinases suggest co-evolution of the RNAP II C-terminal domain and CTD-directed CDKs

**DOI:** 10.1186/1471-2164-5-69

**Published:** 2004-09-20

**Authors:** Zhenhua Guo, John W Stiller

**Affiliations:** 1 Department of Biology, East Carolina University, Howell Science Complex N 108, Greenville, NC 27858, USA

## Abstract

**Background:**

Cyclin-dependent kinases (CDKs) are a large family of proteins that function in a variety of key regulatory pathways in eukaryotic cells, including control over the cell cycle and gene transcription. Among the most important and broadly studied of these roles is reversible phosphorylation of the C-terminal domain (CTD) of RNA polymerase II, part of a complex array of CTD/protein interactions that coordinate the RNAP II transcription cycle. The RNAP CTD is strongly conserved in some groups of eukaryotes, but highly degenerate or absent in others; the reasons for these differences in stabilizing selection on CTD structure are not clear. Given the importance of reversible phosphorylation for CTD-based transcription, the distribution and evolutionary history of CDKs may be a key to understanding differences in constraints on CTD structure; however, the origins and evolutionary relationships of CTD kinases have not been investigated thoroughly. Moreover, although the functions of most CDKs are reasonably well studied in mammals and yeasts, very little is known from most other eukaryotes.

**Results:**

Here we identify 123 CDK family members from animals, plants, yeasts, and four protists from which genome sequences have been completed, and 10 additional CDKs from incomplete genome sequences of organisms with known CTD sequences. Comparative genomic and phylogenetic analyses suggest that cell-cycle CDKs are present in all organisms sampled in this study. In contrast, no clear orthologs of transcription-related CDKs are identified in the most putatively ancestral eukaryotes, *Trypanosoma *or *Giardia*. Kinases involved in CTD phosphorylation, CDK7, CDK8 and CDK9, all are recovered as well-supported and distinct orthologous families, but their relationships to each other and other CDKs are not well-resolved. Significantly, clear orthologs of CDK7 and CDK8 are restricted to only those organisms belonging to groups in which the RNAP II CTD is strongly conserved.

**Conclusions:**

The apparent origins of CDK7 and CDK8, or at least their conservation as clearly recognizable orthologous families, correlate with strong stabilizing selection on RNAP II CTD structure. This suggests co-evolution of the CTD and these CTD-directed CDKs. This observation is consistent with the hypothesis that CDK7 and CDK8 originated at about the same time that the CTD was canalized as the staging platform RNAP II transcription. Alternatively, extensive CTD phosphorylation may occur in only a subset of eukaryotes and, when present, this interaction results in greater stabilizing selection on both CTD and CDK sequences. Overall, our results suggest that transcription-related kinases originated after cell-cycle related CDKs, and became more evolutionarily and functionally diverse as transcriptional complexity increased.

## Background

Cyclin-dependent kinases (CDKs) belong to a large protein family with 13 members described so far in human cells including CDKs1-11, along with PCTAIRE and PFTAIRE kinases named after conserved domain sequences [1]. These kinases are essential for cell cycle progression, and also are involved in control of transcription, DNA repair and post-mitotic cellular process [2-4]. Generally, CDKs1-6, PCTAIRE and PFTAIRE have been linked to cell cycle regulation, and CDKs7, 8 and 9 to control of RNA polymerase II (RNAP II) transcription [4-8]. The functions of CDKs10 and 11 have not been defined clearly, but recent research implicates them in coordination of transcription and RNA-processing [9-13].

Among the most important and broadly studied roles of CDKs in transcription is the reversible phosphorylation of the C-terminal domain (CTD) of the largest subunit (RPB1) of RNAP II. The CTD consists of multiple repeats of an evolutionarily conserved heptapeptide with the consensus sequence Tyr_1_-Ser_2_-Pro_3_-Thr_4_-Ser_5_-Pro_6_-Ser_7 _[14]. The number of repeats varies among different organisms, ranging from 26–27 in yeast to 52 in mammals [15,16] with 8 repeats in yeast and 28 repeats in human cells required for viability [15,17,18]. Both biochemical and genetic evidence places the CTD in a central position in the 'mRNA factory,' where it functions as a platform for interactions with processing factors and other transcription-related proteins [19,20]. More than a passive scaffold, reversible phosphorylation of the CTD regulates the cycling of RNAP II between a hypophosphorylated (IIO) form, which is competent to enter the preinitiation complex, and a hyperphosphorylated (IIA) form capable of processive transcript elongation [21]. Throughout this cycle the CTD binds essential transcription-related proteins that help to regulate gene expression, promote efficient elongation, and effectively couple transcription to pre-mRNA processing [19-24].

To date at least five of the CDKs (CDK1, 2, 7, 8 and 9) have been shown to phosphorylate the CTD *in vitro*; they all have been referred to as 'CTD kinases' [25-28]. Both CDK7 and CDK8 are found tightly associated with the pre-initiation complex and are involved in transcriptional regulation [29]. The CDK9 subunit of P-TEFb (positive transcription elongation factor b) induces hyper-phosphorylation of the CTD and stimulates elongation. Unlike CDKs 7, 8 and 9, which have demonstrated interactions with the CTD *in vivo*, CDK1 and CDK2 are primarily cell-cycle related kinases [4]. CDK2 has been characterized functionally only human and *Drosophila *in mammals and its role in Tat-dependent HIV-1 transcription is still unclear [27,28]. Although phosphorylation of yeast RNAP II by CDK1 (CDC2) can inhibit transcription *in vitro*, the role of the CDK1 in mRNA synthesis *in vivo *is not, as yet, clearly understood. It has been proposed as a candidate for mitotic RNAP II inactivation by inhibition of CDK7 CTD-kinase activity [26].

In animals and yeasts, interactions between the CTD and CTD-specific kinases have become a focal point of biochemical and genetic investigations of RNAP II transcription and transcription-linked mRNA processing [25,26,30]. However, the ancestry and evolutionary relationships among CTD kinases have not been investigated thoroughly. Evolutionary analyses of the RNAP II CTD show that canonical CTD heptads are strongly conserved only in a subset of eukaryotic groups. In evolutionary trees based on RPB1 sequences, all eukaryotic groups in which the CTD is strongly conserved appear to be descended from a single common ancestor (descendents of this ancestor have been referred to as the "CTD-clade") [31]. The reasons for differential conservation of the CTD have not been clarified, nor have evolutionary correlations been established between strong conservation of CTD structure and the presence of essential CTD/protein interactions. In addition, although the functions of various CDKs are reasonably well characterized in mammals and yeasts, very little is known for most other eukaryotes, and the overall evolution of CDKs has been investigated only in animals and yeasts [32]. Therefore, a comparative evolutionary study also can provide clues as to which CDK orthologs, and presumably CDK functions, are present over a broad range of eukaryotic diversity.

Here we present a comparative genomic analysis of CDKs, using complete genomes from members of the "CTD clade" (animals, plants, yeasts and Microsporidia), as well as from other diverse eukaryotic organisms lacking a canonical CTD (*Trypanosoma*, *Plasmodium *and *Giardia*), to explore the evolutionary relationships between the CTD and CTD kinases. We also provide a phylogenetic distribution of CDKs from a wide range of organisms, suggesting new hypotheses regarding the emergence and evolution of different members of the CDK family.

## Results

**Figure 1:**
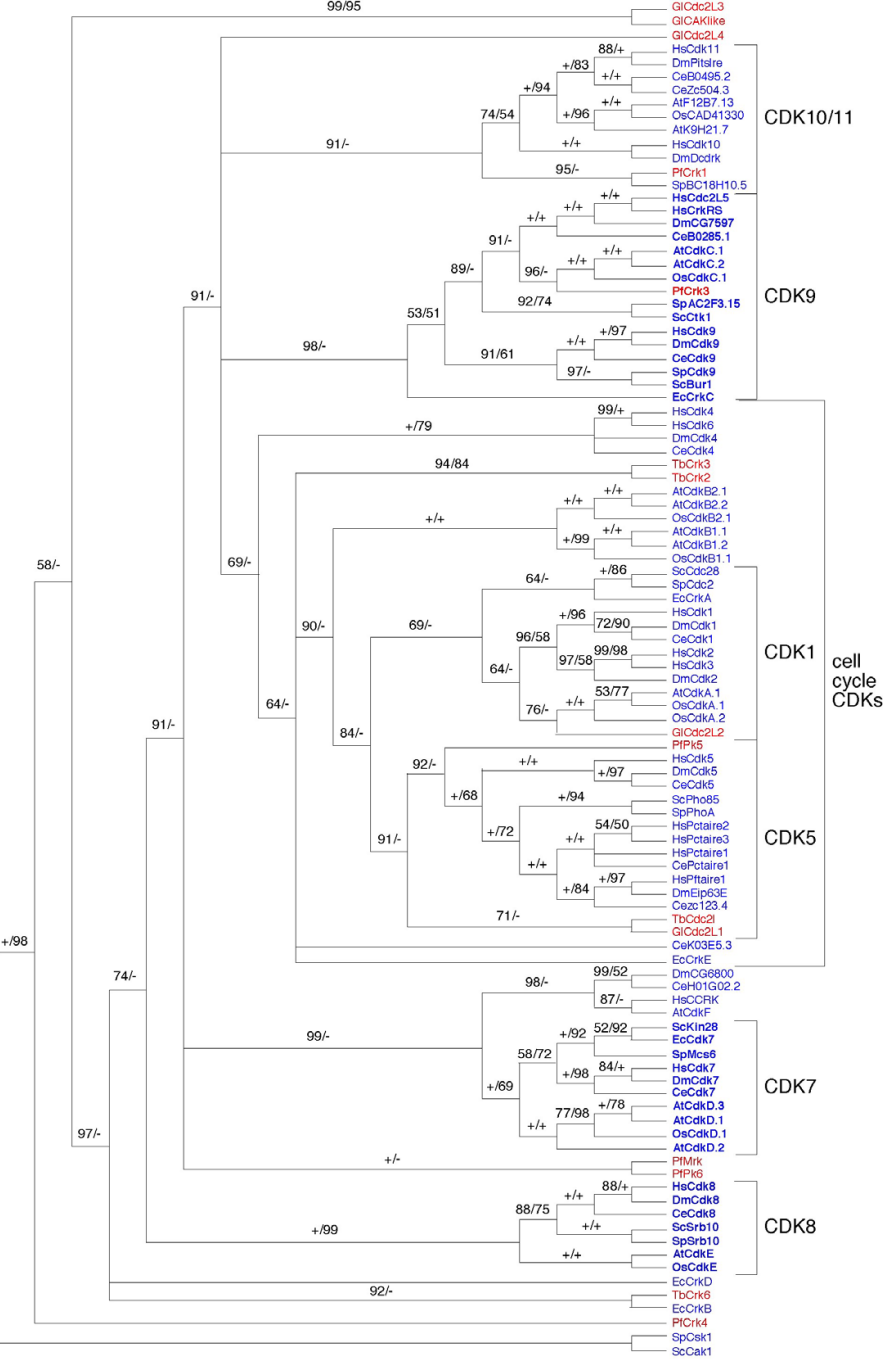
Unrooted 50% majority consensus tree from 4,000 ML trees sampled from the Bayesian posterior probability distribution. Support values are shown above the internode from Bayesian inference/distance bootstrap respectively. Only values above 50% are reported and values under 50% are indicated by (-). 100% values are indicated by (+). CDK names in blue are from organisms that fall into the "CTD-clade" in RPB1 phylogenetic analyses (see Fig. 2); and those in red are from groups in which the CTD is not strongly conserved. Inferred groups of CTD-directed CDKs 7, 8 and 9 are shown in bold. A large group of unidentified CDKs from *Arabidopsis *and *Oryza*, which appear to represent a plant-specific amplification of CTK9, were excluded from this analysis to determine whether identified plant CDK9s show a specific phylogenetic affinity to either the BUR1 or CTK1 subgroup. All identified plant sequences are included in an expanded analysis shown in additional file 1.

We identified 133 CDK family members, 123 from animals, plants, yeasts, and four protists from which genome sequences have been completed, and 10 additional CDKs from incomplete genome sequences of organisms with known CTD sequences (Table 1). Although all of sequences are included in our supplemental phylogenetic analysis (additional file 1), only 101 of them are included in the major phylogenetic analysis (Fig. 1); a large plant-specific amplification of CDK9-like kinases (the phylogenetic weight of these sequences disrupts the CDK9 sub-clade) and sequences from incomplete genomes are excluded (see Fig. 1 and additional file 1 legends for further explanation). The nomenclature for kinases from *Arabidopsis *followed Joubès et al. (2000) and Vandepoele et al. (2002) [33,34] (Table 1). The catalytic core base, Gly-rich motif and T-loop, required for characterized CDK function, appear to be conserved across all defined and putative kinase sequences analyzed (additional file 2). The 50% majority rule consensus tree of 4,000 likelihood trees, sampled from the posterior probability distribution from Bayesian phylogenetic inference, is shown in Figure 1. This tree provides strong support for grouping a number of previously uncharacterized CDKs, from a variety of organisms, with defined CDKs from animals and yeast. Overall, however, very little support is found for relationships among different CDK orthologous groups.

**Table 1:** CDK-related kinases used in this study.

Species	Genes	gi number	Abbreviations
*Trypanosoma brucei*	Cdc2-related kinase2	397162	TbCrk2
	Cdc2-related kinase3	397365	TbCrk3
	Cdc2-related kinase6	23392965	TbCrk6
	Cdc2-like kinase	10458	TbCdc2L
*Leishmania major*	**Cdc2-related kinase 1**	**9857049**	**LmCrk1**
	**Cdc2-related kinase 3**	**15526337**	**LmCrk3**
*Giardia lamblia*	Cdc2-like1	29248279	GlCdc2L1
	Cdc2-like2	29245850	GlCdc2L2
	Cdc2-like3	29250990	GlCdc2L3
	Cdc2-like4	29249431	GlCdc2L4
	CAKlike	29249713	GlCAKlike
*Cryptosporidium parvum*	**Cdc2-like kinase**	**3329529**	**CpCdc2L**
*Plasmodium falciparum*	MO15-related kinase	23507945	PfMrk
	PK5	23619490	PfPk5
	PK6	23618947	PfPk6
	Crk1	23510162	PfCrk1
	Crk3	23509994	PfCrk3
	Crk4	23957709	PfCrk4
*Dictyostelium discoideum*	**Cdc2 kinase**	**167686**	**DdCdc2**
	**Cdc2-related protein**	**167696**	**DdCrp**
	**Cdk7**	**1705721**	**DdCdk7**
	**Cdk8**	**15778146**	**DdCdk8**
	**Cdk9-like kinase**	**28828850**	**DdCdk9L**
*Entamoeba histolytica*	**Cdc2 kinase**	**543971**	**EhCdc2**
*Guillardia theta*	**Cdc2 kinase**	**13812042**	**GtCdc2**
*Saccharomyces cerevisiae*	Cdc28	115915	ScCdc28
	Pho85	295932	ScPho85
	Kin28	1199540	ScKin28
	Cdk8/Srb10	2131219	ScSrb10
	Ctk1	486235	ScCtk1
	Bur1	218486	ScBur1
	Cak1	1480663	ScCak1
*Schizosaccharomyces pombe*	Cdc2	173359	SpCdc2
	PhoA	19075421	SpPhoA
	Mcs6	19113141	SpMcs6
	Cdk8/Srb10	7493197	SpSrb10
	AC2F3.15	19115305	SpAC2F3.15
	Cdk9	32363142	SpCdk9
	Csk1	299548	SpCsk1
	BC18H10.5	3006177	SpBC18H10.5
*Encephalitozoon cuniculi*	Cdc2-related kinaseA	19173516	EcCrkA
	Cdc2-related kinaseB	19069621	EcCrkB
	Cdc2-related kinaseC	19171093	EcCrkC
	Cdc2-related kinaseD	19074929	EcCrkD
	Cdc2-related kinaseE	19173349	EcCrkE
	Cdk7 like kinase	19068706	EcCdk7
*Drosophila melanogaster*	Cdk1	115921	DmCdk1
	Cdc2c	7708	DmCdk2
	Cdk4	1523997	DmCdk4
	Cdk5	1523999	DmCdk5
	Cdk7	1336061	DmCdk7
	Cdk8	1718193	DmCdk8
	Cdk9	24658274	DmCdk9
	Dcdrk	541654	DmDcdrk
	CG6800	23171908	DmCG6800
	Pitslre	1524005	DmPitslre
	CG7597	24668136	DmCG7597
	EiP63E	1524003	DmEip63E
*Caenorhabditis elegans*	K03E5.3	3158523	CeK03E5.3
	Cdk1	5001728	CeCdk1
	Cdk4	21902501	CeCdk4
	Cdk5	5001732	CeCdk5
	Zc123.4	21913082	CeZc123.4
	Pctaire1	5001730	CePctaire1
	Cdc2-like kinase5	7494824	CeB0385.1
	Cdk7	5031478	CeCdk7
	Cdk8	32563668	CeCdk8
	Cdk9	17507939	CeCdk9
	B0495.2	2499649	CeB0495.2
	Zc504.3	897712	CeZc504.3
	H01G02.2	7504821	CeH01G02.2
*Homo sapiens*	Cdk1	115922	HsCdk1
	Cdk2	29849	HsCdk2
	Cdk3	4557439	HsCdk3
	Cdk4	33304135	HsCdk4
	Cdk5	7434324	HsCdk5
	Cdk6	21885467	HsCdk6
	Pctaire1	13623189	HsPctaire1
	Pctaire2	21542571	HsPctaire2
	Pctaire3	30583437	HsPctaire3
	Pftaire1	6912584	HsPftaire1
	Cdk7	13529020	HsCdk7
	Cdk8	1000491	HsCdk8
	Cdk9	12805029	HsCdk9
	Cdk10	6226784	HsCdk10
	Cdk11	16357492	HsCdk11
	Cdc2-Like kinase5	10443222	HsCdc2L5
	Cdc2-related kinase with RS domain	7107392	HsCrkRS
	Cell cycle related kinase	23344742	HsCCRK
*Oryza sativa*	CdkA.1	20343	OsCdkA.1
	CdkA.2	266410	OsCdkA.2
	CdkB2.1	7489567	OsCdkB2.1
	CdkB1.1	34907628	OsCdkB1.1
	R2	231707	OsCdk7
	CdkE	12039362	OsCdkE
	CdkC.1	31442141	OsCdkC.1
	OJ991113_30.14	38344237	OsCAD41330
	**B1015E06.16**	**34903661**	**OsB1015E06.16**
	**P0560B06.11**	**34914693**	**OsP0560B06.11**
	**P0453E05.113**	**28460677**	**OsP0453E05.113**
	**P0450A04.129**	**34899281**	**OsP0450A04.129**
	**P0498H04.21**	**42408343**	**OsP0498H04.21**
	**P0435E12.11**	**46390990**	**OsP0435E12.11**
	**P0482D04.8**	**34907029**	**OsP0482D04.8**
	**OJ1562.H01.5**	**38424086**	**Os1562.H01.5**
*Arabidopsis thaliana*	CdkA1	30693081	AtCdkA.1
	CdkB1.1	30694007	AtCdkB1.1
	CdkB1.2	42569740	AtCdkB1.2
	CdkB2.1	30699181	AtCdkB2.1
	CdkB2.2	18394928	AtCdkB2.2
	CAK1	15235518	AtCdkF
	CAK2	15147864	AtCdkD.3
	CAK3	15147866	AtCdkD.1
	CAK4	20521156	AtCdkD.2
	CdkE	10177042	AtCdkE
	CdkC.1	30698081	AtCdkC.1
	CdkC.2	11346412	AtCdkC.2
	F12B7.13	17065202	AtF12B7.13
	K9H21.7	17064770	AtK9H21.7
	**K9L2.5**	**15241455**	**AtK9L2.5**
	**T22H22.5**	**25405751**	**AtT22H22.5**
	**T12H1.1**	**15229881**	**AtT12H1.1**
	**K16E14.2**	**26449318**	**AtK16E14.2**
	**F21B7.1**	**7488248**	**AtF21B7.1**
	**AT4g22940**	**15235867**	**At4g22940**
	**F8L10.9**	**15219169**	**AtF8L10.9**
	**F26A9.10**	**42572067**	**AtF26A9.10**
	**AT4g10010**	**30681286**	**At4g10010**
	**F14J9.26**	**18391043**	**AtF14J9.26**
	**F6A14.22**	**15221833**	**AtF6A14.22**
	**F1M20.1**	**25406336**	**AtF1M20.1**
	**AAF21469.1**	**6649591**	**AtAAF21469.1**
	**T4P13.34**	**42570106**	**AtT4P13.34**

Note: The sequences in bold are the additional sequences from incomplete genomes and uncharacterized CDK9 like-kinases from *Arabidopsis *and *Oryza *included in supplemental phylogenetic tree (additional file 1).

In this unrooted tree the highly diversified cell-cycle kinases defined in humans, CDKs1-6, fall into a large cluster with 69% Bayesian support. This grouping includes CDKs from all organisms examined in the study. Among these putative cell-cycle CDKs, some plant and protistan kinases can be assigned with reasonable confidence to specific CDK groups. For example, apparent orthologs of human CDK1 are found in other animals (*Drosophila *and *Caenorhabditis*), yeasts, both plants (*Arabidopsis *and *Oryza*), *Encephalitozoon *and *Giardia *(Fig. 1). Likewise, putative orthologs of CDK5 were identified in all organisms examined, except for the two plants (Fig. 1). A number of other sequences, such as TbCrk2 and 3 from *Trypanosoma*, cluster with cell-cycle kinases but not clearly with any specific CDK family. Significantly, and consistent with the results of Liu and Kipreos (2000) [32], CDK5 and PCTAIRE-like kinases from fungi and animals form a strongly supported group, indicating their close relationship (Fig. 1).

**Figure 2:**
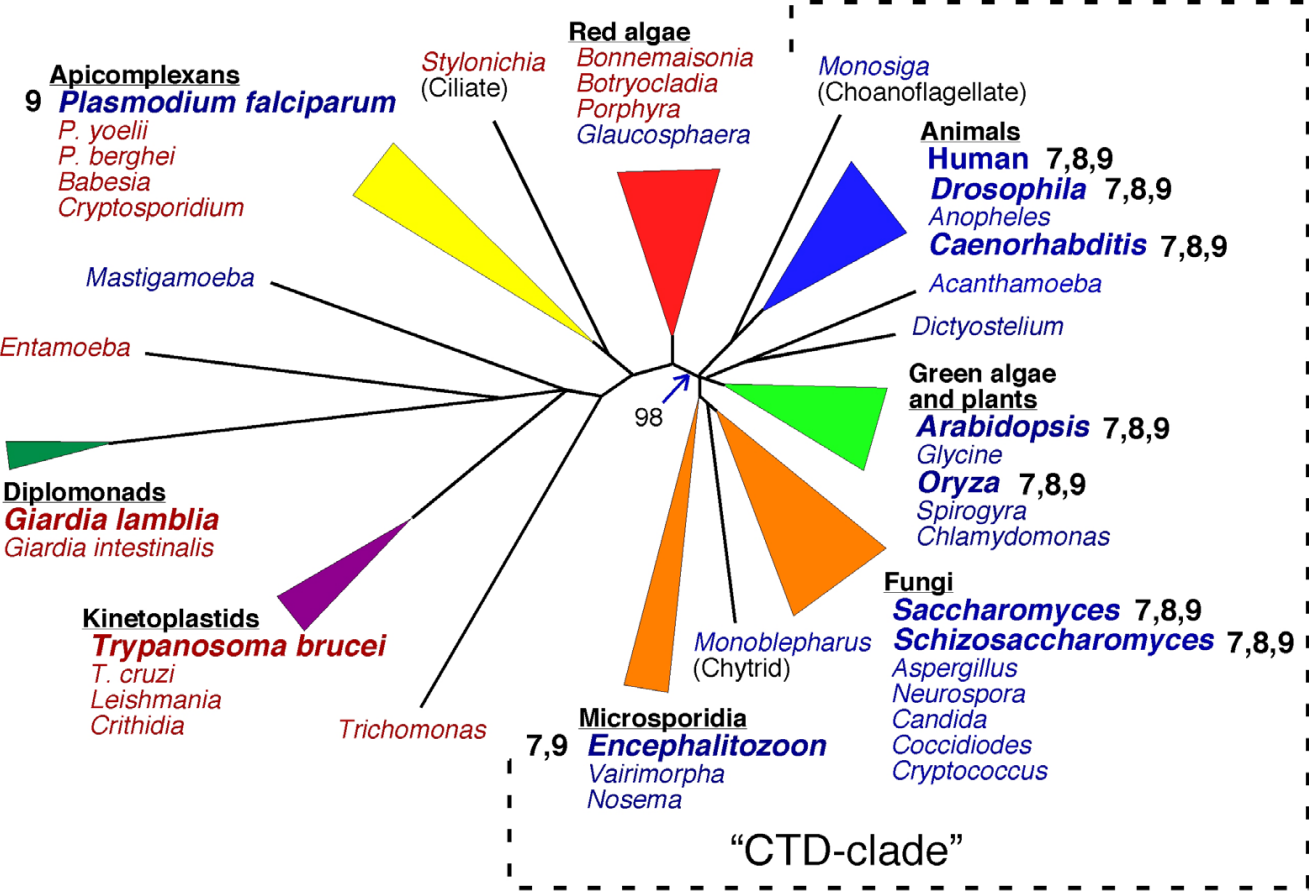
Hypothesis of RNA polymerase II evolution inferred from phylogenetic analyses of RPB1 sequences conserved regions A-H. The tree displayed, after Stiller and Cook [60] had the highest likelihood of all trees sampled from the posterior probability distribution in 10^6 ^generations of Bayesian inference. Organisms with genomes included in this study are in larger/bold font, and whether each of the three primary CTD kinases (CDKs7,8,9) are present in this genome, as inferred from global phylogenetic analyses and distributions of CDK kinases (see Fig. 1), are indicated next to the name. Names in blue indicate the presence of repeated heptads at the RPB1 C-terminus, which includes several from protistan organisms that do not conform to the consensus sequence or known structural requirements of the canonical CTD [60]; names in red have no tandem-heptapeptide structure whatsoever. The node supporting a "CTD-clade," in which the consensus sequence and repetitive structure of the CTD are invariably conserved, occurred in 98% of the 8000 trees sampled from the Bayesian posterior probability distribution. See references 31 and 60 for a more complete phylogenetic treatment of the origin and conservation of the CTD.

In contrast to cell-cycle kinases, our phylogenetic results failed to identify a clear ortholog of any transcription-related CDKs from two of the complete genomes examined, *Trypanosoma brucei *and *Giardia lamblia*. This includes strongly supported clades of presumed orthologs of human CDKs7-11 respectively. A well-defined CDK7 family is recovered, including sequences from yeasts, the microsporidian, plants, and animals. These are the primary groups that make up the "CTD-clade," in which the RNAP II CTD is invariably conserved (Fig. 2). CDK7 shows an interesting sister relationship to HsCCRK from human and apparent orthologs from *Drosophila*, *Caeorhabditis *and *Arabidopsis*. In *Arabidopsis*, four possible CDK7 orthologs were found, as reported previously by Shimotohno and colleagues (2003) [35]; however, AtCdkF (CAK1) is quite divergent from the core CDK7 family and related specifically to HsCCRK in our analyses. PfMRK from *Plasmodium*, suggested previously to be a CDK7 [36], does not fall within the well-defined CDK7 group, but clusters with another *Plasmodium *kinase. The *a priori *hypothesis that PfMRK belongs in the core CDK7 group is strongly rejected with our data set in a likelihood paired-sites test.

Likewise, GlCAKlike (gi: 292497120) has been proposed as a CDK7 from *Giardia*, based on nearest sequence similarity to Kin28 in a more limited comparison to CDK sequences from fission yeast [38]. In our expanded analyses of CDKs from 11 completed genomes, we find no evidence supporting an orthologous relationship to CDK7 for this, or any *Giardia *sequence. The *a priori *hypothesis that GlCAKlike belongs in the core CDK7 group also is strongly rejected in a likelihood paired-sites test.

A robust CDK8 family is recovered with strong support values in both distance bootstrap and Bayesian inference. Like CDK7, this family includes putative orthologs only from members of the "CTD-clade," specifically yeasts, animals and plants. Although the microsporidian *Encephalitozoon *is a member of the RNAP II "CTD clade," TBlastN searches of the complete genome of *Encephalitozoon *found six CDKs but none show a phylogenetic affinity to CDK8.

A CDK9 grouping also is supported as monophyletic with representative CDKs from yeasts, *Encephalitozoon*, animals, plants and *Plasmodium*. This group is divided into two well-defined sub-clades. One of them consists of BUR1 from yeast along with CDK9 orthologs from animals; the other contains CTK1 from yeast, CDC2L5 and CrkRS from human, and apparent orthologs from *Drosophila *and *Caenorhabditis*, both plants, and *Plasmodium*. A putative CDK9 also is found in *Encephalitozoon*, but falls at the base of the larger CDK9 grouping and does not associate clearly with either subgroup (Fig. 1). Plants also contain a large number of putative CDKs that show strong phylogenetic affinity to CDK9 (additional file 1). These kinases appear to represent a plant-specific amplification of CDK9, although their functions have not been determined experimentally.

Human CDK10 and CDK11 group with apparent orthologs from other animals, plants, fission yeast, and PfCRK1 from *Plasmodium*. Once again, no kinases from either *Trypanosoma *or *Giardia *show any phylogenetic affinity to this group.

## Discussion

### A suggestion of co-evolution between the RNAP II CTD and CTD kinases

At least five CDKs have the capacity to phosphorylate RNAP II CTD repeats *in vitro *or *in vivo *[25-28]. Kinases that appear to be related closely to CDK1, which can phosphorylate the CTD *in vitro*, are present in all organisms sampled; however, it is not clear that CDK1 interacts with the CTD *in vivo *or is essential for CTD function. CDK2 was found only in human and *Drosophila *and, based on our analyses from a diverse group of eukaryotes, appears to be derived from within a larger CDK1 family. In any case, according to its restricted phylogenetic distribution, possible CTD/CDK2 interactions cannot explain the conservation of CTD structure in diverse members of the "CTD-clade."

Evolutionary investigations of RPB1 sequences show that canonical CTD heptads are conserved strongly in only a subset of eukaryotic groups, all apparently descended from a single common ancestor [31]. This "CTD-clade" is composed of animals, plants, fungi, and related protistan groups, including microsporidians, chytridiomycetes, choanoflagellates and slime molds (Fig. 2). A handful of organisms that do not fall inside the "CTD-clade" do have tandemly repeated C-terminal heptads. For example, RPB1 from *Plasmodium falciparum *contains a short set of seven tandem C-terminal repeats. Based on codon usage and comparative alignment with sequences from other *Plasmodium *species, these heptads are best explained by a recent tandem duplication of a single heptad motif in *P. falciparum *or its immediate ancestor [31]. No other apicomplexan RPB1 contains tandemly repeated heptads, nor does the nearest evolutionary relative of the apicomplexans (Fig. 2). Although vestigial or convergent heptad repeats are found in a few organisms scattered across the eukaryotic evolutionary tree, strong stabilizing selection on CTD structure appears to be restricted to those eukaryotic lineages found in the "CTD-clade" (Fig. 2).

In our analyses of CDKs, members of this "CTD-clade" are precisely the same eukaryotes to which clear orthologs of CDK7 and CDK8 are restricted. When sequences recovered from additional but incomplete eukaryotic genomes are included in phylogenetic analyses, distribution of these two kinases remains tightly correlated with strong conservation of canonical CTD repeats (see additional file 1). Moreover, unlike CDK1, the primary characterized function of both of these kinases is to mediate RNAP IIA/IIO cycling through reversible phosphorylation of CTD residues [19-24].

Taken together, these findings suggest that the RNAP II CTD has undergone a co-evolutionary process with CDK7 and CDK8. If phylogenetic results based on CDK and RPB1 sequences reflect evolutionary history, the inference of a "CTD-clade" in both sets of analyses suggests that CDK7 and 8 originated as part of a major shift in the mechanics of RNAP II transcription in the ancestor of the "CTD-clade" [31]. It was in that ancestor that reversible phosphorylation of the CTD became a central organizing principle for regulating the transcription cycle, and laid the foundation for more complicated mechanisms of transcriptional control in these organisms. Such a profound shift in the mechanics of RNAP II transcription would explain why the CTD is conserved so strongly in members of the CTD-clade, but not in many other eukaryotic lineages [31]. In this scenario, other known or putative CTD kinases (certainly CDK1 and apparently CDK9) originated before canalization of a CTD-based RNAP II transcription cycle, and were adapted later as CTD kinases.

It also is possible that the co-evolution inferred from comparisons of the phylogenetic distribution of RPB1 and CDKs7/8 does not reflect the pattern of evolutionary history but, instead, results from functional constraints driven by CTD/CDK interactions. Both GlCAKlike from *Giardia *and PfMrk from *Plasmodium *have been suggested previously to be orthologs of CDK7 [36,38]; these hypotheses are rejected strongly by our phylogenetic analyses. Assuming these kinases really are CDK7s, then their failure to cluster with other orthologs must be due to phylogenetic artifacts, frequently referred to as "long-branch attraction" [39], that can be common when rates of evolution vary dramatically among sequences. The large amounts of sequence divergence of PfMrk and GlCAKlike from other CDK7s, along with a complete degeneration of the CTD in *Giardia *species and apicomplexans as a group, are unlikely to coincidental. It is possible that those organisms retaining a RNAP II transcription cycle mediated by CDK7 and 8 kinase activity form distinct clades, in both RPB1 and kinase derived trees, because both sets of proteins share parallel modes of evolution driven by their physical interactions. In this case, the observation of co-evolution between the CTD and CTD-directed kinases need not have a phylogenetic basis, only a functional one.

Most putative CDKs from *Giardia *and *Trypanosoma*, and several from *Plasmodium*, do not associate strongly with any established CDK family. It is reasonable to assume that at least some of these kinases are orthologs of defined CDK groups, but have diverged to the point that they are not recognizable using sequence-based phylogenetic methods. Although such a scenario may have disturbing implications for the use of these methods across broad evolutionary distances, particularly when functional interactions among sequences are unknown or poorly understood, it cannot be ruled out as an explanation for our observations. Analyses of additional genomes from diverse eukaryotes are required, both to verify our observations of co-evolution between the CTD and CTD-directed kinases, and to determine its bases.

### General evolutionary trends in the CDK family

#### Kinases from protistan organisms

In an effort to understand the broader evolutionary history of CDKs, three deep-branching protists with complete genomes, *Plasmodium falciparum*, *Trypanosoma brucei *and *Giardia lamblia*, were included in our study. Our Blast searches detected 15 putative kinases from these protists; six from *Plasmodium falciparum*, four from *Trypanosoma brucei *and five from *Giardia lamblia *(Table 1). The phylogenetic positions and orthologous relationships of these kinases generally are not well defined by phylogenetic analyses (Fig. 1). Four of them (GlCdc2L3, GlCAKlike, TbCrk6 and PfCrk4), along with two microsporidian kinases (EcCrkB and EcCrkD) branched close to ScCak1 and SpCsk1, cyclin-activating kinases from yeasts. All of these sequences are highly divergent, and it is difficult to determine, whether their branching positions are due to a phylogenetic artifact or a phylogenetic relationship. As noted above, GlCAKlike kinase has been proposed as a *Giardia *CDK7 ortholog based on JTT distance data [38], a relationship not supported by our broader phylogenetic analyses. Moreover, there are no experimental data reported on the functions of any of these kinases. Other putative protistan CDKs, GlCdc2L4, PfMrk and PfPk6, scatter among CDKs from other organisms, but with no statistical confidence for any implied relationship. Our most strongly supported results indicate that six of these kinases (TbCdc2L, TbCrk2 and 3, PfPk5, GlCdc2L1 and L2) belong to cell-cycle related kinase families CDK1 and CDK5. In particular, PfPk5 is well-supported as an ortholog of CDK5. In addition, two kinases from *Plasmodium *(PfCrk1 and PfCrk3) appear to be transcription-related kinases, PfCrk1 groups with the CDK10/11 family, and PfCrk3 with CDK9.

The phylogenetic distribution of protistan kinases indicate that cell-cycle related kinases are present, or at least their functions are more strongly conserved (see discussion above regarding CTD/CDK co-evolution), in a more diverse array of eukaryotes than are transcription-related kinases. This pattern also is seen in a more widely-sampled analyses including CDKs from a number of organisms with incompletely sequenced genomes, including *Dictyostelium discoideum *that has a canonical RNAP II CTD, and *Leishmania major*, *Cryptosporidium parvum *and *Entamoeba histolytica*, which all lack a CTD (see additional file 1). Thus, the overall results suggest that cell-cycle related kinases are more ancient than transcription-related kinases, and probably ancestral to them, and that their core functions are more similar across the broad diversity of eukaryotic lineages. It will be interesting to see whether these preliminary hypotheses are supported as more genomes are sequenced completely, particularly from diverse protistan organisms.

#### Cell-cycle related kinases

Our analyses support well-defined groups for cell-cycle kinases CDK1, CDK4/6 and CDK5. An ortholog of either CDK1 or CDK5 is found in all of the organisms in our study, and these two families appear to be closely related. TbCrk3 was proposed as a functional homolog of CDK1 in *Trypanosoma *[40]; here it groups among cell-cycle kinases, but is not specifically related to CDK1. CDK4/6 appears to be present only in human, *Drosophila *and *C. elegans*. The CDK5 family has undergone expansion in metazoans, including PFTAIRE and PCTAIRE kinases, and putative orthologs of CDK5 are detected in *Plasmodium*, *Trypanosoma *and *Giardia*. Interestingly, no CDK from plants associates strongly with the CDK5 group, while the CdkB-type kinases, which are specific to plants, branch as sister to a broader CDK1/CDK5 clade. Our overall results suggest that cell-cycle kinases have undergone extensive and independent evolutionary diversification in different eukaryotic lineages, and it may be difficult to classify many of them based on orthologous relationships in phylogenetic analyses. It may be that functional homologies, once established experimentally, will prove to be more consistent criteria for designating CDK groups.

#### The CDK7 family

Clear orthologs of CDK7 from animals, plants, yeasts and Microsporidian are strongly supported as a core family, with CDK-activating kinase from *Arabidopsis *(AtCdkF), and its apparent orthologs from animals, branching as a sister group. In addition to their role as CTD kinases, members of the CDK7 family in plants, animals and fission yeast can function as a CDK-activating kinase (CAK) [41,42]. Unlike animals and yeast, however, four CDK7-like of CAKs were isolated from *Arabidopsis *[35]. AtCdkF (AtCAK1), which groups with human CCRK and apparent orthologs from *Drosophila *and *Caenorhabiditis*, exhibits only CAK activity but no CTD kinase activity. Consistent with the phylogenetic relationships recovered in our analysis, human CCRK and other animal orthologs were recently shown to have CAK activity [43]. In contrast, AtCdkD3 (AtCAK2) and AtCdkD2 (AtCAK4) display both CAK and CTD kinase activity and, along with a single CDK7 from rice, are included in a strongly supported CDK7 clade. Interestingly, and despite its high sequence similarity to AtCdkD3, no kinase activity was reported from AtCdkD1 (AtCAK3) [35]. Apparently CAKs in *Arabidopsis *have diversified substantially, and may be regulated in different ways from those in yeast, animals, and even rice.

ScCAK1 and SpCSK1 from yeasts also have CAK activity; however, despite their functional similarity to kinases in the CCRK group, they do not group with animal or plant CAKs (Fig. 1). Interestingly, in the single most likely tree recovered in our expanded Bayesian analysis of 133 sequences, ScCAK1 and SpCsk1 group with other CAKs in the sister clade to CDK7 (additional file 1); however, there is no support for this placement in the Bayesian probability distribution. ScCAK1 and SpCSK1 sequences are highly divergent from all CDKs, and the regulation of CAK activity in yeast is very different from that of animals and plants [42,44]. Thus, alternative lines of evidence may be required to determine whether there is any specific evolutionary relationship among all CAKs.

#### The CDK 8 family

CDK 8 (SRB10 in yeast) is a component of the multi-subunit Mediator complex, which transduces signals from cis regulatory elements to RNAP II; it is proposed to inhibit transcription initiation by phosphorylation of the CTD. CDK8/SRB10 and its partner cyclin C/SRB11, together with SRB8 and SRB9, form a specific sub-module that is variably associated with the RNAP II holoenzyme, and potentially with the free mediator complex [45]. Apparent orthologs of CDK8 form a well-defined group, including sequences from plants, animals and yeasts. Interestingly, although a member of the CTD clade (Fig. 2 and note that all microsporidian RPB1 genes isolated to date encode a CTD), no ortholog of CDK8 was identified from *Encephalitozoon*. Our further blast results (unpublished data) failed to identify any of the units of the CDK8/SRB10 (SRBs8-11) sub-module in the Microsporidia suggesting a loss of CDK8/SRB10 unit from these highly reduced parasites.

Although the CDK8/SRB10 sub-module has been implicated in negative regulation of transcription by phosphorylation of TFIIH, leading to the inhibition of the TFIIH CTD kinase and transcription [46], the exact mechanism still is unclear. Recent research shows that the Mediator containing this sub-module is isolated only in free form, not associated with RNAP II. In contrast, Mediator lacking this sub-module associates with the polymerase [47]. There also is experimental evidence that negative Mediator-RNAP II regulation by the SRB8-11 sub-module is evolutionarily conserved from yeast to humans [47]. Therefore, the absence of identifiable components of the SRB8-11 sub-module in *Encephalitozoon *suggests CDK8/SRB10 function is absent from the Microsporidia. The loss of CDK8 from Microsporidia, along with absolute conservation of CDKs7 and 9 in all members of the "CTD-clade" (Figs. 1 and 2) implies that interactions between the CTD and Mediator complex are less strongly entrained into essential RNAP II function, than are those regulated by TFIIH and P-TEFB kinase activity.

#### The CDK 9 family

CDK9 is a component of the P-TEFb complex, which is a positive-acting RNAP II transcription elongation factor [48,49]. Research has focused on P-TEFb from animals and budding yeast. A definitive yeast homolog of animal P-TEFb has not yet been determined from functional studies, but two candidates have emerged: the BUR1 complex and the CTDK-I complex [26]. Based on our blast and phylogenetic analyses, BUR1 and CTK1 (subunit of CTDK-I complex) are found in two distinct but related kinase groups, each with orthologs from other eukaryotes. BUR1 is identified as the specific ortholog of CDK9 from metazoans, budding yeast and probably the Microsporidia.

Unexpectedly, the CDC2-like5 kinases and CrkRS from animals are highly supported as orthologs of CTK1 from yeasts. Although their functions are not yet clear [50], our results suggest that human CDC2-like5 kinases and CrkRS have CDK9 function. Recent analyses of CrkRS (CDC2-related kinase with an RS-rich domain) suggest that it has CTD kinase activity and helps to link transcription directly to intron splicing [51]. This CTK1 clade also contains putative CDK9 (CdkC) kinases from plants and as well as a CDC2-like kinase from *Plasmodium *(PfCRK3). The latter is the only apparent ortholog of a CTD-directed kinase (CDKs 7, 8 or 9) identified in our analyses from any organism outside the "CTD-clade." It remains to be determined whether PfCRK3 possesses the P-TEFb function of CTK1, since it is the only protistan sequence present in either CDK9 sub-group, and the RNAP II CTD has not been conserved in apicomplexans or their closest relatives (Fig. 2).

In addition to the two previously identified copies of CDK9 (CdkC1 and CdkC2) from *Arabidopsis*, and one from *Oryza *(CdkC1) [33,34], our Blast searches also retrieved a large group of CDK9-like sequences (14 from *Arabidopsis *and 8 from *Oryza*) (Table 1). These kinases are annotated as "Cdc2-like" in databases and some of them also were identified in previous analyses of CDK evolution [38]. With one exception (Os1562.H01.5), all of these kinases group in a single cluster, with 100% support, and as sister to previously identified CDK9s of *Arabidopsis *and *Oryza *(additional file 1). Os1562.H01.5 (Gi: 38424086) from *Oryza *is extremely similar to OsCdkC1 and very likely a second copy of CdkC (CDK9) from *Oryza*. There is no evidence of biological functions for these kinases as yet, but our results indicate that they are part of a large CDK9 complex specific to plants.

#### The CDK10/11 family

In this group, orthologs of CDK10 are found only in human and *Drosophila*, while CDK11 occurs in human, *Drosophila *and *Caenorhabditis*. Three putative CDK11 orthologs were found in plants (two from *Arabidopsis *and one from *Oryza*). CDK10 has been implicated in the regulation of the G2/M phase of the cell cycle [52], but a cyclin partner has yet to be defined. Only one protein associated with CDK10, ETS2 transcription factor, has been identified so far, suggesting a link to transcription [9]. CDK11 associates with cyclin L as a partner, and is a proposed component of a signaling pathway that helps to coordinate transcription and RNA-processing events [10-13]. The close relationship between the CDK10 and CDK11 may reflect evolutionary and/or mechanistic similarities, but neither kinase family has been well characterized functionally. In addition, BC18H10 from *S. pombe *and PfCRK1 from *Plasmodium *show close relationships to the CDK10/11 family, but no function has yet been determined for these kinases either.

## Conclusions

The apparent co-evolution between the CTD and certain CTD-specific kinases suggests an explanation for strong stabilizing selection on CTD structure in some eukaryotes, and its complete degeneration in others. Based on the genomes examined in this study, either the origins of CDK7 and CDK8 in an unknown ancestor of the "CTD-clade," or the canalization of reversible phosphorylation of the CTD in some eukaryotic groups but not others, could account for the variation seen in RPB1 C-terminal structure. In either case, once thoroughly "locked" into RNAP II function, the CTD must have recruited other transcription and processing related proteins into a growing machinery of the "transcriptosome" [53]. Our results suggest that was the case for several CDKs that clearly predate the canalization of CTD-based RNAP II transcription; further genomic analyses are underway to look for other protein-protein interactions that could be responsible for strong evolutionary conservation of the CTD in members of the "CTD-clade."

This work also provides a new perspective on the overall evolution CDKs and evolutionary relationships among kinase families. Our combined genomic and phylogenetic analyses suggest that transcription-related kinases originated later than cell cycle-related CDKs. Finally, our results point to potential functions for a variety of previously uncharacterized kinases, based on their apparent orthologous relationships to defined CDKs. Additional completed genomes, particularly those from broadly diverse protists (especially non-parasitic forms), will be critical to address these questions further. Such comparative analyses will be invaluable in helping to guide experimental studies, which ultimately are required to verify the functional properties of each putative CDK.

## Methods

### Identification and alignment of protein sequences

Representatives of all previously identified CDKs from budding yeast and human were obtained from Genbank, and used as probes in TBlastN and PSI-Blast [54] against the National Center for Biotechnology Information (NCBI), and additional specific complete genome databases, with an absolute cut-off of E<0.001. To confirm the identities of putative CDKs detected by the TBlastN, each identified sequence was used as a query in reciprocal Blast searches, to verify that it retrieved the original query sequences, and global sequence alignments were performed to confirm putative homologies to CDKs, according to the CDC-related kinase characterized motifs that use CDK2 as the model [55].

Initially, a number of inferred protein sequences were grouped into six subsets according to clear similarities to specific CDK family orthologs. These subgroups first were aligned in CLUSTAL X [56], and the resulting sub-alignments then were aligned with each other and adjusted through visual inspection and comparison to the kinase alignment of Liu and Kipreos (2000) [32]. Regions that could not be aligned reliably were excluded from subsequent phylogenetic analysis. The resulting alignment included 233 positions including gaps (See additional data file 2 and 3 for the original and final aligned matrices used in this study).

### Phylogenetic analysis

Maximum-likelihood (ML) estimates of substitution parameters were made with the program TREEPUZZLE-50 [57] assuming a mixed model for variation among sites, with one category for invariable sites and a four-category discrete approximation to Γ-distribution, and the JTT weighting matrix for probability of change among amino acids. Further analyses were performed in MrBayes 3.0 b4 [58] using metropolis-coupled Markov chain Monte Carlo analysis. Four simultaneous Markov chains were run, also under an invariant + Γ rate model and a JTT substitution matrix. Four chains, one heated, were run for 500,000 generations, beginning with random *a priori *trees. Trees were sampled from the posterior probability distribution every 100 generations. The empirical burn-in required for likelihoods to converge was less than 100,000 generations; an additional 400,000 generations were run and the first 100,000 were excluded from analysis of Bayesian posterior probabilities. Thus, a total of 4,000 trees were examined to determine the 50% majority-rule consensus tree and Bayesian support values. In addition, 1000 distance (PROTDIST + NEIGHBOR) bootstrap replicates were performed in PHYLIP 3.573 [59], also using a JTT substitution model.

Several *a priori *alternative hypotheses regarding CDK7 evolution were compared by KHT likelihood paired-sites tests [37]. Trees were constrained to require PfMRK from *Plasmodium *or GlCAKlike from *Giardia*, which previously have been characterized as a CDK7 orthologs [36,38], to group with the well-defined CDK7 clade. All most parsimonious trees retaining these constrained relationships were tested against the fully resolved Bayesian consensus tree to determine whether the *a priori *hypotheses of orthologous relationships to CDK7 were significantly worse than the Bayesian consensus tree.

## Authors' contributions

ZG was primarily responsible for database searching and assembly of CDK genes. ZG and JWS performed phylogenetic analysis. ZG drafted the manuscript and figures and JWS contributed editorial revisions. All authors read and approved the final manuscript.

## Supplementary Material

Additional File 1 The single most likely tree, with branch lengths, recovered from 16,000 ML trees in the posterior probability distributions of four separate iterations of Bayesian inference. Thirty-two additional sequences were added to this analysis, and are indicated in bold in Table 1. They represent CDKs identified in incomplete genomes of organisms from which CTD structure is known, as well as a large amplification of apparent plant-specific orthologs of CDK9 from *Arabidopsis *and *Oryza*. The phylogenetic weight of these latter plant sequences disrupts inferred relationships among CDK9 orthologs as shown in Fig. 1. Support values are from Bayesian inference and only values above 50% are shown. As in Fig. 1, CDK names in red are from groups in which the CTD is not strongly conserved, those in blue from members of the "CTD-clade." Inferred groups of CTD-directed CDKs 7, 8 and 9 are shown on the tree. Click here for file

Additional File 2 Original protein sequence alignment. Click here for file

Additional File 3 Edited protein sequence alignment. Click here for file
