# Supplementary figures and images for: Comparative genomics of cyclin-dependent kinases suggest co-evolution of the RNAP II C-terminal domain and CTD-directed CDKs
Source: BMC Genomics. 2004 Sep 20;5:69. doi: 10.1186/1471-2164-5-69 (PMC521075; doi:10.1186/1471-2164-5-69)

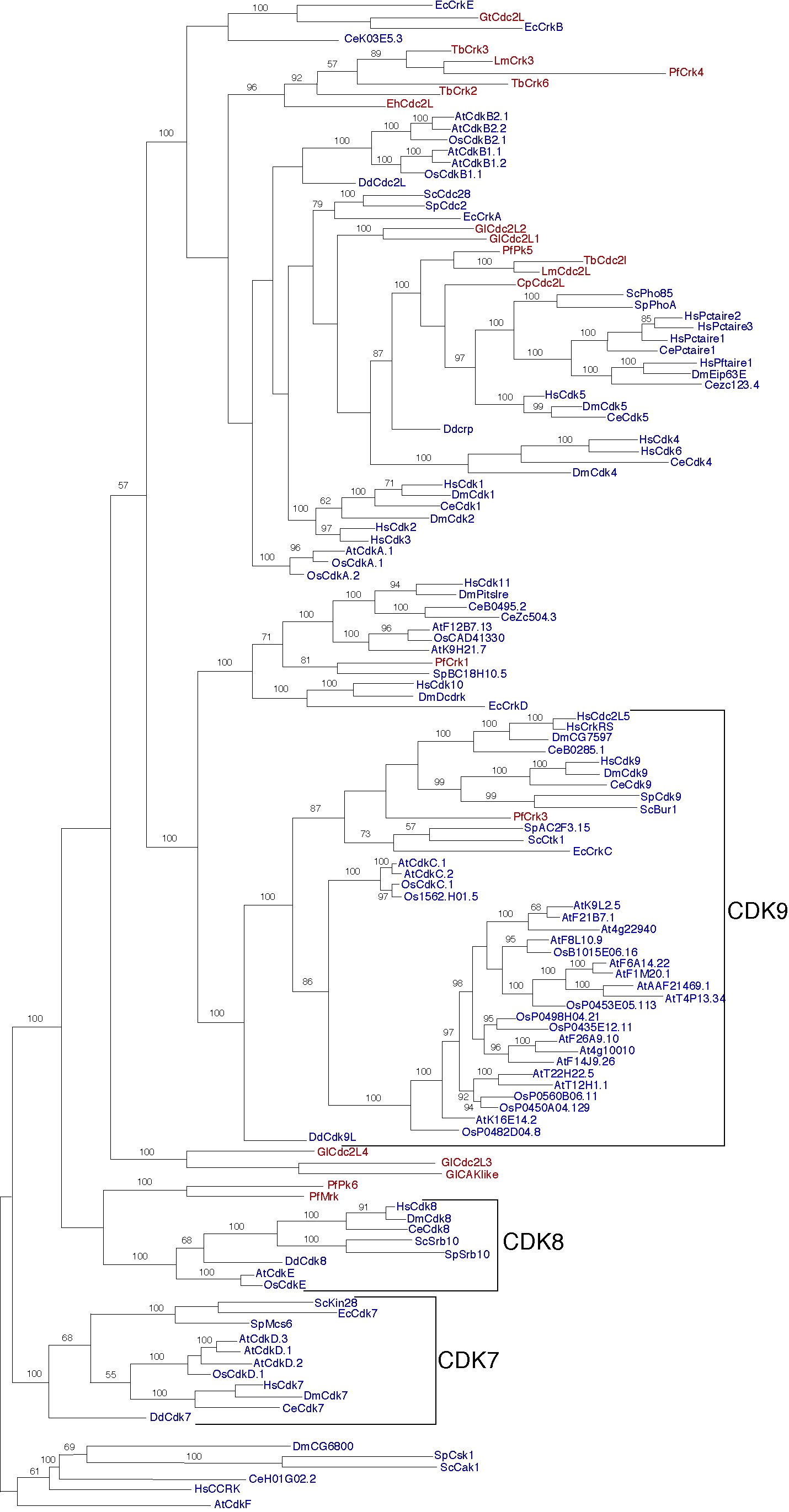

Supplement: Additional File 1 — The single most likely tree, with branch lengths, recovered from 16,000 ML trees in the posterior probability distributions of four separate iterations of Bayesian inference. Thirty-two additional sequences were added to this analysis, and are indicated in bold in Table 1. They represent CDKs identified in incomplete genomes of organisms from which CTD structure is known, as well as a large amplification of apparent plant-specific orthologs of CDK9 from Arabidopsis and Oryza. The phylogenetic weight of these latter plant sequences disrupts inferred relationships among CDK9 orthologs as shown in Fig. 1. Support values are from Bayesian inference and only values above 50% are shown. As in Fig. 1, CDK names in red are from groups in which the CTD is not strongly conserved, those in blue from members of the "CTD-clade." Inferred groups of CTD-directed CDKs 7, 8 and 9 are shown on the tree. [file 1471-2164-5-69-S1.jpeg]
